# Insecticidal and Repellent Activity of *Piper crassinervium* Essential Oil and Its Pure Compounds Against Imported Fire Ants (Hymenoptera: Formicidae)

**DOI:** 10.3390/molecules29225430

**Published:** 2024-11-18

**Authors:** Farhan Mahmood Shah, Mei Wang, Jianping Zhao, Joseph Lee, Paulo Vitor Farago, Jane Manfron, Ikhlas A. Khan, Abbas Ali

**Affiliations:** 1 National Center for Natural Products Research, School of Pharmacy, University of Mississippi, University, MS 38677, USA; 2 Natural Products Utilization Research Unit, Agricultural Research Service, United States Department of Agriculture, University, MS 38677, USA; 3 Postgraduate Program in Pharmaceutical Sciences, State University of Ponta Grossa, Ponta Grossa 84030-900, PR, Brazil

**Keywords:** *Piper crassinervium*, essential oil, imported fire ants, repellency, toxicity, digging behavior

## Abstract

*Piper crassinervium* Kunth (Piperaceae) essential oil (EO) was evaluated for its toxicity and repellency against red imported fire ants (RIFA), *Solenopsis invicta* Buren, and a hybrid (HIFA) of red (*S. invicta)* and black (*S. richteri* Forel) imported fire ants. Through bioactivity-guided fractionation, two major components, elemicin and myristicin, were isolated from the EO. Removal of treated sand in a digging bioassay was used as the criterion for repellency. The EO showed significantly higher repellency at concentrations of 7.8 µg/g against RIFA and HIFA workers, as compared to the DEET (*N*,*N*-diethyl-*meta*-toluamide) or ethanol control. Elemicin exhibited repellency at 3.9 and 7.8 µg/g against RIFA and HIFA workers, respectively, whereas myristicin was active at 7.8 µg/g against both species. DEET failed at 31.25 µg/g against RIFA and 15.6 µg/g against HIFA. The EO showed LC_50_ values of 97.9 and 73.7 µg/g against RIFA and HIFA workers, respectively. Myristicin was more toxic against RIFA and HIFA with LC_50_ values of 54.3 and 35.3 µg/g, respectively. Elemicin showed 20–40% mortality at the highest screening dose of 125 µg/g. Fipronil exhibited the highest toxicity against RIFA and HIFA, with LC_50_ of 0.43 and 0.51 µg/g, respectively. Different formulations of these natural products should be evaluated to explore their use potential under natural field conditions.

## 1. Introduction

Red imported fire ants (RIFA), *Solenopsis invicta* Buren, and black imported fire ants (BIFA), *S. richteri* Forel (Formicidae: Hymenoptera), have become widespread in the southeastern United States, parts of western states, Mexico, and Puerto Rico [1] after introduction from their native South America in the early 1900s. Their reproductively functional hybrids, *S. invicta* × *S. richteri* (HIFA), are also widespread in the states of Alabama, Arkansas, Georgia, Mississippi, and Tennessee. Imported fire ant populations escalate rapidly due to their aggressive behavior, strong competitive ability, high reproductive capacity, and omnivorous diets [1,2,3,4]. Increasing populations pose serious risks to human health, public safety, wildlife, agriculture, forestry, and ecological environments [5,6,7,8], resulting in $6.7 billion in annual losses incurred in the United States [9]. To help limit the further spread of imported fire ants, a federal quarantine law (7 CFR 301.81) has been implemented by the United States. Specifically, RIFA is a serious concern as an invasive species that exhibits a wider geographic range adaptability [10,11].

Current methods used for the management of imported fire ants include applications of toxic baits and synthetic insecticides through individual mound treatments [12,13]. These methods undoubtedly provide rapid control; however, maintaining control over ant populations may require repeated use. The repeated, long-term use of these chemical methods can complicate their control by triggering ant populations to adapt behaviorally (e.g., particle covering behavior and post-treatment mound relocation) or use detoxification mechanisms to avoid toxicants [14,15,16,17]. The overuse of pesticides can also threaten human and environmental health. By contrast, natural products, being environmentally friendly alternatives, are gaining increased attention on the development of novel preventive tools for use in sustainable pest management programs [18,19]. Due to their high species specificity and affinity, natural products are generally non-toxic [20] and are considered safe to use in limiting the spread of imported fire ants [21,22,23].

Our natural products screening research program is focused on exploring plant-derived repellents and toxicants with the potential of being used against imported fire ants [24,25]. Phytochemicals, including plant extracts and essential oils (EOs), offer novel chemistries to be explored for their potential as pesticides [26,27]. The bioactive compounds from natural products may act as attractants, antifeedants, or oviposition modifiers, or they may affect key metabolic processes, leading to rapid death. Plant metabolites, such as monoterpenoids, are neurotoxic and cause death by disrupting neurotransmission [28,29]. Terpenoids, such as callicarpenal and intermedeol from *Callicarpa americana* L. and *Callicarpa japonica* Thunb. exhibited repellency against imported fire ants [30]. In digging bioassays, the ant workers’ ability to remove sand was significantly reduced when exposed to the sand treated with *Magnolia grandiflora* L. (Magnoliaceae) EO, its pure components [31], and *Matricaria chamomilla* L., (Asteraceae) [32]. He et al. [33] reported that treating flowerpot sand with methyl isoeugenol prevented RIFA workers from nesting in those pots one month post treatment. By negatively affecting digging/nesting activities, the probability of nest construction or colony establishment may be hampered [34,35].

The genus *Piper* in the plant family Piperaceae is widely distributed in tropical and subtropical regions of Africa, America, and Asia. The genus includes approximately 700 species of herbs, shrubs, and trees, many of which are aromatic and are used in folk medicine and for isolation of biologically diverse compounds including secondary metabolites (alkaloids, flavonoids, and terpenes) with insecticidal activity [36,37,38]. *Piper crassinervium* Kunth is one such plant that exhibits insecticidal activity against *Drosophila suzukii* Matsumura (Diptera: Drosophilidae) [39] and *Aedes aegypti* (L.) (Diptera: Culicidae) [40]. This plant species has never been evaluated against imported fire ants. The present study reports the repellency and toxicity of *P. crassinervium* EO and its two active components against red and hybrid imported fire ants.

## 2. Results

### 2.1. Chemical Composition of EO

The chemical profile of *P. crassinervium* EO was determined by GC-MS analysis, and the total ion chromatogram is presented in Supplementary Figure S1. A total of 56 prominent compounds, each constituting more than 0.1% of the peak area, were identified, collectively representing 91.49% of the composition. Identification was achieved through a combination of methods, including MS spectra matching with a NIST library search, comparison with reference standards, and determination of the relative retention index (RRI) by referring to relevant literature data (Table 1 for details). The main constituents present in *P. crassinervium* EO were myristicin (33.55%) followed by elemicin (9.63%), *epi*-β-caryophyllene (7.20%), β-caryophyllene (4.32%), α-muurolene (3.50%), γ-muurolene (3.22%), and spathulenol (2.85%). In contrast, a previous study by Cysne et al. [41] reported that the major compound in *P. crassinervium* EO was linalool (28.61%), followed by β-pinene (20.01%), α-pinene (11.27%), and 1,8-cineole (10.81%). After the initial screening, the EO showed toxicity and repellency against imported fire ants, then chromatographic fractionation of the EO was conducted by using a silica gel column. Elution was carried out utilizing a hexane–acetyl acetate solvent system with a gradual increase in polarity from 0 to 60% acetyl acetate. Fractions were collected and subsequently combined based on TLC and GC-MS analyses, resulting in the generation of 6 fractions (Frs. 7–8, Fr. 9, Frs. 10–12, Frs. 29–33, Frs. 34–40, and Frs. 41–48). The EO and its fractions were then tested with the bioassay.

### 2.2. Digging Bioassay

Mean weight (g) of treated sand removed by HIFA and RIFA workers is presented in Table 2. *Piper crassinervium* EO exhibited significantly greater repellency than ethanol control at concentrations of 7.8 µg/g against both RIFA and HIFA. The two fractions, Frs. 7–8 and Fr. 9, exhibited significantly higher repellency against HIFA at 15.6 µg/g as compared to the ethanol control. Through bioassay-guided fractionation, two pure components, namely elemicin and myristicin, were isolated from Fr. 7–8 and Fr. 9, respectively. Further digging bioassay tests showed that elemicin was repellent at 3.9 and 7.8 µg/g against RIFA and HIFA, respectively, and myristicin was active at 7.8 µg/g against the workers of both species. In DEET treatments, repellency was significantly greater than the ethanol control at concentrations of 62.5 against RIFA and 31.25 µg/g against HIFA. Natural products showed significantly higher repellency than DEET against imported fire ants.

### 2.3. Toxicity Bioassay

*Piper crassinervium* EO, its isolated compounds, and fipronil were evaluated to determine their dose–response of toxicity against RIFA and HIFA workers (Table 3). *Piper crassinervium* EO showed toxicity against RIFA and HIFA workers with LC_50_ values of 97.9 and 73.7 µg/g, respectively. Fraction Fr.9 showed toxicity with LC_50_ values of 50.5 and 53.4 µg/g against RIFA and HIFA workers, respectively. Among *P. crassinervium* EO, fractions, and isolated compounds, myristicin was the most active against RIFA and HIFA, with LC_50_ values of 43.4 and 31.3 µg/g, respectively, whereas fipronil showed toxicity against RIFA and HIFA with LC_50_ of 0.43 and 0.51 µg/g, respectively.

## 3. Discussion

*Piper crassinervium* EO is a significant source of bioactive compounds, exhibiting a diverse range of therapeutic effects. Nevertheless, research on the phytochemicals responsible for these biological impacts, such as their efficacy in repelling fire ants, remains scarce. As part of our ongoing efforts to assess natural products for their biological activity against imported fire ants, this study was designed to evaluate the toxicity and repellency of *P. crassinervium* EO against imported fire ants. The EO showed toxicity and repellency, based on which it was further explored to determine its chemical composition and to identify the components responsible for the activity.

The EO and phytochemical constituents of *Piper* species have been shown to exhibit diverse biological activities against insect arthropods. The main mechanism of action is neurotoxicity which affects insects by acting as an acetylcholinesterase inhibitor where prolonged muscle contraction leads to paralysis and death [38,43,44]. In addition to killing the larvae, EOs of *P. purusanum* C.DC with β-caryophyllene (57.05%), α-humulene (14.50%), and germacrene D (8.20%) as the main constituents, were shown to significantly suppress egg hatching in *Aedes* and *Anopheles* mosquitoes [38]. Using ingestion and topical application bioassays, de Souza et al. [39] found that the EOs from *P. aduncum* L., *P. gaudichaudianum* Kunth, and *P. marginatum* Jacq. caused 100% mortality of adults of *Drosophila suzukii* (Diptera: Drosophilidae), whereas the dry residues of these EOs affected oviposition and egg viability. Monoterpene hydrocarbons (5.3–60.9%), oxygenated monoterpenes (13.3%), sesquiterpenes hydrocarbons (8.3–45.3%), oxygenated sesquiterpenes (5.2–58.8%), and arylpropanoids (15.2–29.6%) were the major components in those EOs. The EOs from leaves of *P. holtonii* C.DC. and *P. reticulatum* L. exhibited significantly higher repellency than chlorpyrifos at 4 h against *Sitophilus zeamais* Motschulsky (Coleoptera: Curculionidae) [45]. The repellent action of EO from the leaves of *P. aduncum*, containing nerolidol, α-humulene, and β-caryophyllene as its major components, was reported against *Tetranychus urticae* Koch. (Acari: Tetranychidae) [46]. *Piper aduncum* EO was proven to be a potential repellent against *Ae. aegypti* as its treatment on human skin prevented mosquito bites [47]. The EO of *P. yunnanense* Y.C. Tseng fruit, with γ-muurolene as its major component, demonstrated remarkable repellency at 78.63 nL/cm^2^ against *Tribolium castaneum* (Herbst) (Coleoptera: Tenebrionidae) at 2 and 4 h post exposure [48]. Bhoopong et al. [49] reported contact irritancy of mace extract of *Myristica fragrans* Houtt. (Magnoliales: Myristicaceae), containing elemicin (11.68%) and myristicin (9.76%) as the primary chemical constituents, against *Aedes* mosquitoes with an escape response of 28.07% at a concentration of 5.0% (*w*/*v*). The literature on the repellent activities of *P. crassinervium* EO against fire ants is scarce. The data from the present study demonstrated that *P. crassinervium* EO and its pure components have the potential to be developed as a natural repellent against imported fire ants. Based on our repellency data, the pure compounds elemicin and myristicin appear to be the main compounds responsible for repellency of this EO. The significantly higher repellency of natural products than DEET corroborates the findings of Paudel et al. [50] and Shah et al. [32]. Since treatment with repellents can negatively affect worker digging behavior, the use of these natural products may be potentially suitable in preventing ants from invading sensitive areas, nursery stock, and equipment as quarantine treatments. This is the first report of repellency of *P. crassinervium* EO and the pure compounds elemicin and myristicin against imported fire ants.

The toxicity of *Piper* species EOs has been reported against many species of insects. de Souza et al. [39] reported the toxicity of *P. crassinervium* EO against *D. suzukii* adults using the ingestion and topical application bioassays. *Piper crassinervium* EO, with α-pinene, β-pinene, and β-caryophyllene as its major components, showed toxicity against a pyrethroid resistant strain of *Ae. aegypti* larvae [40]. In another report, the EO from the leaves of *P. purusanum,* with β-caryophyllene (57.05%), α-humulene (14.50%), and germacrene D (8.20%) as its major components, exhibited toxicity against larvae of malaria and dengue mosquitoes by inhibiting the action of acetylcholinesterase [38]. In vitro trypanocidal activity of the major compound prenylated hydroquinone extracted from leaves of *P. crassinervium* was reported against *Trypanosoma cruzi* (Y strain), with the IC_50_ value of 6.10 µg mL^−1^ compared with the positive control benznidazole (IC_50_ = 1.60 µg/mL) [51]. Contact mortality against stored grain pests for the EOs of *P. yunnanense,* against *T. castaneum* (35.84 μg/adult) and *Liposceis bostrychophila* (57.70 μg/cm^2^), and *P. boehmeriifolium* (Miq.) Wall. ex C.DC., against *Lasioderma serricorne* (15.76 μg/adult), was reported [48]. The literature on the toxicity of *P. crassinervium* EO against fire ants is scarce. Souto et al. [36] reported contact toxicity of the EOs from *P. aduncum*, *P. marginatum* (chemotypes A and B), *P. divaricatum* G.Mey., and *P. callosum* Ruiz & Pav. against the fire ants *Solenopsis saevissima* (Smith) (Hymenoptera: Formicidae). The EOs exhibited contact toxicity with LC_50s_ of 58.4 mg/L for *P. aduncum*, 122.4 mg/L for *P. marginatum* chemotype A, 167.0 mg/L for *P. marginatum* chemotype B, 301.7 mg/L for *P. divaricatum*, and 312.6 mg/L for *P. callosum* with dillapiole (64.4%), *p*-mentha-1(7),8-diene (39.0%), (*E*)-isoosmorhizole (32.2%), methyl eugenol (69.2%), and safrole (69.2%) as the major components, respectively. We found that the EO of *P. crassinervium* and its major component myristicin were more toxic against HIFA than RIFA, and the toxicity varied among ant species and was dose dependent. Myristicin was more toxic than the EO suggesting that this main compound is responsible for toxicity, as elemicin caused very low mortality in fire ants. Data from this study corroborate the findings of Almadiy et al. [52], who identified myristicin and elemicin from the EO of *Deverra tortuosa* (Desf.) DC. (Apiaceae) and reported higher contact mortality of myristicin than elemicin against *Callosobruchus maculatus* F. (Coleoptera: Chrysomelidae). Dorla et al. [53] reported that the contact toxicity of the EO from *Peperomia borbonensis* MIQ. (Piperaceae) leaves against *Bactrocera cucurbitae* (Diptera: Tephritidae) was primarily attributed to the synergistic effect of its main constituents, myristicin and elemicin, as the individual activity of these compounds was significantly lower than that of their combined mixture. Based on our experience with fire ants, we anticipate that contact toxicity and repellency work together, with the repellency impairing digging ability and the toxicity eliminating the workers that enter the treated areas. Since these compounds have the ability to suppress worker digging ability, treating individual mounds by drenching or preventive spraying in lawns may reduce the risk of imported fire ant infestation in the treated areas. This is the first report of toxicity of *P. crassinervium* EO and its main components against imported fire ants.

While the active components elemicin and myristicin are important as food and natural medicine treatments and as biopesticides in plant protection, adverse health impacts or toxicity have been associated with their high dose consumption [54,55]. Therefore, they are considered to be safe but higher dosages may produce negative effects. However, the toxicity profile should be determined before the use of these compounds as pesticides.

## 4. Materials and Methods

### 4.1. Materials

DEET (CAS # 134-62-3) and fipronil (CAS #120068-37-3) were purchased from Sigma-Aldrich (Saint Louis, MO, USA). The reference standards listed in Table 1 for compound identification, including α-pinene (98%), camphene (>96%), β-pinene (98.5%), limonene (>99%), β-elemene (>98%), α-gurjunene (>97%), β-caryophyllene (>80%), caryophyllene oxide (95%), and viridiflorol (95%) were supplied by Sigma-Aldrich. Analytical grade 3-carene and *epi*-cedrol were obtained from Agilent Technologies (Santa Clara, CA, USA). α-Humulene was obtained from Cayman Chemical (Ann Arbor, MI, USA), while myristicin, elemicin, and spathulenol were isolated in-house. The identities and purities of these compounds were confirmed using spectral data (NMR and HRMS). The purities of myristicin, elemicin, and spathulenol were determined to be 95%, 92%, and 85%, respectively.

### 4.2. Plant Material

Fresh leaf samples of *P. crassinervium* were collected in January 2022 from open, sun-exposed habitats in Antonina, Paraná, Southern Brazil (coordinates 25°28′34′′ S, 48°41′07′′ W). The specimens were taxonomically identified, and a voucher specimen has been deposited in the Museu Botânico de Curitiba under the accession number MBM 306,102 (Curitiba, Paraná, Brazil). Collection and access to the botanical material was authorized and licensed by the Conselho de Gestão do Patrimônio Genético (CGEN/SISGEN), with registration code AC1A4A.

### 4.3. Extraction of EO

The EO was extracted from dried leaves by hydrodistillation. The yield (m/m) of the EO was 0.11%.

### 4.4. GC-MS Analysis

GC-MS analyses were conducted using an Agilent 7890 GC system (Agilent Technologies, Santa Clara, CA, USA). Separation was achieved on an Agilent DB-1MS column (60 m × 0.25 mm × 0.25 µm). Helium was used as the carrier gas, with a constant flow rate of 1 mL/min. The inlet temperature was held at 260 °C in split mode, with a split ratio of 50:1. The GC oven temperature was initially set at 45 °C for 2 min, followed by a temperature ramp of 1.5 °C/min to 140 °C. After reaching 140 °C, the temperature was increased at 6 °C/min until it reached 180 °C.

Mass spectral detection was performed using an Agilent (Santa Clara, CA, USA) 5975C quadrupole mass spectrometer operating in full spectral acquisition mode, scanning from *m/z* 35 to 500. The EI source was set at an energy level of 70 eV. The ion source, quadrupole, and transfer line temperatures were set to 230 °C, 150 °C, and 280 °C, respectively. Tentative compound identification was achieved by comparing the obtained spectra with those in the Wiley and NIST databases using a probability-based matching algorithm [56]. Further identification was based on the relative retention indices compared with the literature [42] and the reference standards procured from commercial sources.

### 4.5. General Experimental Procedures

NMR spectra were acquired on an Agilent DD2-500 NMR spectrometer with a OneNMR probe at 500 MHz for ^1^H and 125 MHz for ^13^C using the pulse programs provided by the Agilent Vnmrj 4.0 software. Silica gel (J. T. Baker, Phillipsburg, NJ, USA, 40 µm for flash chromatography) and Sephadex LH-20 were purchased from Fisher Scientific Co. (Waltham, MA, USA). A Teledyne ISCO flash chromatography system (Lincoln, NE, USA) was used for further separation and purification. TLC was performed on silica gel 60 GF 254 plates (Millipore Sigma, Burlington, MA, USA).

### 4.6. Ants

HIFA workers were directly used from natural mounds at the University Field Station (University of Mississippi, 15 County Road 2078, Abbeville, MS 38601; 34°25′57.2′ N 89°23′25.3′ W). RIFA colonies were brought from Washington County, MS 38,748 (33°09′31.2″ N 90°54′56.4″ W) using escape-proof plastic buckets. The colonies were maintained in their actual mound soil as a digging substrate, by feeding on crickets and 25% sugar-water, for at least one month prior to starting the experiments. The laboratory conditions were set at 32 ± 2 °C temperature; 50% ± 10% relative humidity; 12:12 h (L:D) photophase. The identification of RIFA and BIFA was based on the venom alkaloids and hydrocarbon indices of their workers [31,57].

### 4.7. Digging Bioassay

The repellency of *P. crassinervium* EO and its pure components against imported fire ants was determined using a multiple-choice digging bioassay as described by Chen [58] and Ali et al. [31] (Supplementary Figure S2). In this digging bioassay, worker digging ability is assumed to be an inverse function of repellent treatments, i.e., digging is suppressed by highly effective repellents. Briefly, the bioassay setup consisted of a 150 mm × 15 mm petri dish arena and four 2 mL Nylgene Cryoware Cryogenic vials with lids. The four lids were glued at equal distances on the lower side of the arena and a 3 mm hole was drilled through the base of each lid to provide entry. The inner walls of arena were lined with Insect a Slip. Sand (500 microns) was used as a digging substrate. The sand was washed with deionized water and oven dried at 150 °C for 6 h. Four grams of sand was weighed in 45 mL fluted aluminum weighing dishes and treated in a volume of 400 µL. Both the stock and serial dilutions were prepared in ethanol. After the evaporation of solvent, which required about 10 min under lab conditions, the sand was moistened by adding deionized water at a rate of 0.6 µL/g. The moistened sand was packed tightly in 2 mL vials leaving no spaces. These vials were then screwed to the lids. Control sand was treated with ethanol alone. Each vial had an estimated amount of 3.6 g sand on a dry weight basis. Fifty ant workers were released into the center of the arena petri dish. After 24 h, the left-over sand from the vials was collected back, oven-dried at 180 °C for 1 h, and weighed. *Piper crassinervium* EO and its isolated pure compounds were tested with DEET (*N*,*N*-diethyl-*meta*-toluamide) as a positive control. Fractions were tested against HIFA only because of shortage of samples. A series of serial dilutions with a starting concentration of 125 µg/g were tested until the failure of the treatment. The whole experiment was run in triplicate at different times. Laboratory conditions were set at 32 ± 2 °C temperature and 50 ± 10% relative humidity. Sand removal data were analyzed by Analysis of Variance (ANOVA). Means were separated using the Ryan–Einot–Gabriel–Welsch multiple range test (*p* ≤ 0.05) (SAS 9.4, 2012).

### 4.8. Toxicity Bioassay

The toxicity of *Piper crassinervium* EO and the pure compounds against RIFA and HIFA was determined following the contact bioassay method described by Ali et al. [31]: it is a non-choice bioassay, which assumes that workers dig through when exposed to a suitable substrate; however, mortality occurs in treated sand only at lethal treatments. A series of serial dilutions was tested with fipronil, the active ingredient of many commercially available baits, as a positive control. Both the stock solutions and the dilutions were prepared in ethanol. In 42 mL aluminum fluted dishes, three grams of sand was weighed and treated in a volume of 300 µL. The sand in the controls was treated with ethanol alone. The solvent was evaporated. Deionized water at a rate of 0.6 µL/g was added to moisten the sand. The sand was then transferred into 60 × 15 mm stackable petri dishes, whose inner walls were coated with Insect-a-Slip. Ten workers were released in each petri dish. A water-soaked cotton swab tip was placed in each petri dish to ensure a continuous supply of moisture. Dead ants were counted at 24 h post treatment. The workers that were unable to stand or walk when touched using a hairbrush were also noted as dead. LC_50_ values were calculated using Probit analysis (SAS 2012).

## 5. Conclusions

In conclusion, this study reports the detailed repellency and toxicity of *P. crassinervium* EO and its two major components, myristicin and elemicin, against imported fire ants. Based on digging inhibition as the criterion, the *P. crassinervium* EO and its major components, myristicin and elemicin, showed repellency against imported fire ant workers. Myristicin was significantly more toxic than the EO and appears to be the major compound responsible for the activity. Further studies should be conducted to test different formulations of these natural products to explore their potential for use against invasive fire ants under natural field conditions.

## Figures and Tables

**Table 1:** Chemical profile and tentative compound identification of *P. crassinervium* EO.

No.	RRI^a^	RRI^b^	Compound Name	Relative Peak Area %	Identification Method
1	930	939	α-pinene	1.78	*t_R_*, RRI, MS
2	942	954	camphene	0.37	*t_R_*, RRI, MS
3	969	979	β-pinene	0.66	*t_R_*, RRI, MS
4	1004	1011	3-carene	0.11	*t_R_*, RRI, MS
5	1021	1029	limonene	0.17	*t_R_*, RRI, MS
6	1177	1136	3-caren-10-al	0.10	RRI, MS
7	1324	1338	δ-elemene	0.34	RRI, MS
8	1347	1351	α-cubebene	0.19	RRI, MS
9	1365	1371	cyclosativene	0.16	RRI, MS
10	1374	1376	α-copaene	1.00	RRI, MS
11	1381	1388	β-bourbonene	0.34	RRI, MS
12	1386	1390	β-elemene	1.98	*t_R_*, RRI, MS
13	1397	1398	cyperene	0.13	RRI, MS
14	1407	1409	α-gurjunene	0.58	*t_R_*, RRI, MS
15	1412	1413	β-maaliene	0.24	RRI, MS
16	1416	1419	β-caryophyllene	4.32	*t_R_*, RRI, MS
17	1423	1432	β-copaene	0.19	RRI, MS
18	1428	1433	β-gurjunene	0.10	RRI, MS
19	1430	1434	α-bergamotene	0.30	RRI, MS
20	1435	1443	guaia-6,9-diene	1.35	RRI, MS
21	1446	1454	α-humulene	0.91	*t_R_*, RRI, MS
22	1453	1466	*epi*-β-caryophyllene	7.20	RRI, MS
23	1464	1477	γ-gurjunene	0.23	RRI, MS
24	1467	1479	γ-muurolene	3.22	RRI, MS
25	1472	1481	germacrene-D	2.29	RRI, MS
26	1477	1490	β-eudesmene	0.88	RRI, MS
27	1480	1518	myristicin	33.55	*t_R_*, RRI, MS
28	1481	1522	β-cadinene	0.29	RRI, MS
29	1483	1515	cubebol	0.44	RRI, MS
30	1490	1500	α-muurolene	3.50	RRI, MS
31	1497	1486	eremophilene	0.32	RRI, MS
32	1499	1505	β-bisabolene	0.21	RRI, MS
33	1502	1516	sesquicineole	0.48	RRI, MS
34	1506	1522	calamenene	0.97	RRI, MS
35	1511	1523	cadina-1(10),4-diene	1.71	RRI, MS
36	1515	1557	elemicin	9.63	*t_R_*, RRI, MS
37	1520	1495	cubenene	0.18	RRI, MS
38	1524	1545	α-calacorene	0.17	RRI, MS
39	1529	1549	elemol	0.55	RRI, MS
40	1554	1568	palustrol	0.29	RRI, MS
41	1557	1578	spathulenol	2.85	*t_R_*, RRI, MS
42	1563	1583	caryophyllene oxide	0.80	*t_R_*, RRI, MS
43	1568	1590	globulol	0.34	*t_R_*, RRI, MS
44	1575	1592	viridiflorol	0.22	*t_R_*, RRI, MS
45	1587	1602	ledol	1.21	RRI, MS
46	1610	1619	di-*epi*-1,10-cubenol	0.51	RRI, MS
47	1612	1628	epicubenol	0.36	RRI, MS
48	1615	1619	*epi*-cedrol	0.21	*t_R_*, RRI, MS
49	1620	1642	τ-muurolol	0.42	RRI, MS
50	1623	1654	δ-cadinol	0.53	RRI, MS
51	1625	1646	cubenol	0.22	RRI, MS
52	1632	1654	α-cadinol	0.73	RRI, MS
53	1640	1678	apiol	0.47	RRI, MS
54	1680	1691	alloaromadendrene epoxide	0.86	RRI, MS
55	1714	1709	*cis*-thujopsenal	0.15	RRI, MS
56	1739	1740	oplopanone	0.18	RRI, MS
Total				91.49	

Compound identification methods: *t_R_*, identification based on the retention time (*t_R_*) of genuine compounds on the DB-1MS column; MS, identification based on mass spectra search using Wiley and NIST libraries; RRI, identification based on the comparison of calculated relative retention index with the literature data. RRI^a^: calculated retention index on DB-1MS column relative to *n*-alkanes. RRI^b^, retention index reported in the literature [42].

**Table 2:** Mean weight (g) of treated sand removed by imported fire ant workers released in a multiple-choice digging bioassay with different concentrations of *Piper crassinervium* EO, its fractions, the two pure compounds, and DEET.

Conc. (µg/g)	Mean ± SE ^†^	*F*-Value	*p*-Value	Mean ± SE ^†^	*F*-Value	*p*-Value
	RIFA				HIFA	
*P. crassinervium* EO				-		
Control	1.75 ± 0.06 A	6.39	0.008	2.48 ± 0.03 A	4.61	0.037
15.6	1.23 ± 0.09 B			1.74 ± 0.13 B		
7.8	1.17 ± 0.09 B			1.80 ± 0.26 B		
3.9	1.38 ± 0.14 AB			2.06 ± 0.12 AB		
Frs. 7–8 *						
Control	-			2.08 ± 0.19 A	4.69	0.036
15.6	-			0.73 ± 0.37 B		
7.8	-			1.45 ± 0.21 AB		
3.9	-			1.16 ± 0.23 AB		
Fr. 9 *						
Control	-			1.83 ± 0.22 A	4.01	0.026
15.6	-			0.61 ± 0.20 B		
7.8	-			1.26 ± 0.25 AB		
3.9	-			1.31 ± 0.31 AB		
elemicin						
Control	1.74 ± 0.20 A	1.04	0.423			
1.95	1.49 ± 0.08 A					
0.98	1.58 ± 0.16 A					
0.49	1.33 ± 0.01 A					
Control	1.11 ± 0.20 A	12.17	0.002	1.35 ± 0.16 A	14.11	0.001
15.6	0.08 ± 0.08 B			0.04 ± 0.20 B		
7.8	0.20 ± 0.16 B			0.17 ± 0.17 B		
3.9	0.35 ± 0.01 B			0.83 ± 0.10 A		
myristicin						
Control	1.12 ± 0.21 A	18.34	<0.001	1.38 ± 0.06 A	9.31	0.005
15.6	0.01 ± 0.01 B			0.33 ± 0.11 B		
7.8	0.10 ± 0.05 B			0.61 ± 0.10 B		
3.9	0.77 ± 0.13 A			0.89 ± 0.25 AB		
DEET						
Control	-			1.26 ± 0.19 A	0.24	0.87
15.6	-			0.98 ± 0.49 A		
7.8	-			1.37 ± 0.28 A		
3.9	-			1.16 ± 0.29 A		
Control	1.43 ± 0.19 A	16.24	0.001	1.58 ± 0.11 A	9.71	0.005
125	0.08 ± 0.04 C			0.42 ± 0.25 B		
62.5	0.74 ± 0.18 B			0.87 ± 0.13 B		
31.25	1.14 ± 0.10 AB			0.84 ± 0.04 B		

^†^ Sand removed is in grams. * Fractions were tested only against HIFA because of a shortage of samples. Each individual experiment consisted of three concentrations and a control. Experiments were continued until a failure point was reached. Means within a column in an experiment not followed by the same letter are significantly different (Ryan–Einot–Gabriel–Welsch multiple range test; *p* ≤ 0.05).

**Table 3:** Toxicity of *Piper crassinervium* EO, fractions, and the two pure compounds, against RIFA and HIFA workers at 24 h post treatment.

Compound	*n*	Slope ± SE	LC_50_ (95% CI) *	LC_90_ (95% CI) *	χ^2^	*df*
RIFA						
*P. crassinervium* EO	30	1.23 ± 0.22	97.9 (71.6–142.6)	277.8 (179.9–664.4)	30.644	13
Frs. 7–8 **	30		20%			
Fr. 9	30	1.14 ± 0.27	50.5 (32.7–91.7)	155.3 (87.1–746.1)	17.285	13
myristicin	30	1.04 ± 0.25	43.4 (27.3–81.1)	148.3 (79.9–812.1)	17.076	13
Elemicin **	30		20%			
fipronil	40	0.94 ±0.22	0.43 (0.26–0.81)	1.67 (0.88–7.8)	19.16	26
HIFA						
*P. crassinervium* EO	30	2.83 ± 0.47	73.7 (64.2–85.2)	115.8 (97.9–153.8)	35.734	13
Frs. 7–8 **	30		100%			
Fr. 9	30	1.69 ± 0.24	53.4 (44.8–64.5)	114.1 (89.4–169.6)	46.116	13
myristicin	30	1.77 ± 0.53	31.3 (18.9–57.5)	148.2 (79.7–812.1)	11.13	13
Elemicin **	30		40%			
fipronil	40	1.86 ± 0.33	0.51 (0.4 ± 0.7)	1.02 (0.77 ± 1.7)	33.87	26

* LC_50_ and LC_90_ values are in µg/g and 95% C.I. are confidence intervals. ** Dose–response curves could not be constructed because mortality dropped abruptly to zero from 125 µg/g to the next serial dose at 24 h posttreatment.

## Data Availability

The data presented in this study are available in the article.

## Supplementary Materials

The following supporting information can be downloaded at: https://www.mdpi.com/article/10.3390/molecules29225430/s1, Figure S1: GC/MS total ion chromatogram of *P. crassinervium* EO, Figure S2: Imported fire ants digging bioassay setup (**A**) and digging activity presented as quantity of sand removed as a measure of repellency (**B**).
