# Supplementary material for: Insecticidal and Repellent Activity of Piper crassinervium Essential Oil and Its Pure Compounds Against Imported Fire Ants (Hymenoptera: Formicidae)
Source: Molecules. 2024 Nov 18;29(22):5430. doi: 10.3390/molecules29225430 (PMC11597710; doi:10.3390/molecules29225430)
Supplement: Supplementary file 1 [file molecules-29-05430-s001.zip › molecules-3294009-supplementary.pdf]

Insecticidal and Repellent Activity of *Piper crassinervium* Essential Oil and Its Pure Compounds Against Imported Fire Ants (Hymenoptera: Formicidae)

Farhan Mahmood Shah [1], Mei Wang [2], Jianping Zhao [1], Joseph Lee [1], Paulo Vitor Farago [3], Jane Manfron [3], Ikhlas A. Khan [1] and Abbas Ali [1,*]

[1] National Center for Natural Products Research, School of Pharmacy, University of Mississippi, University, MS 38677, USA
[2] Natural Products Utilization Research Unit, Agricultural Research Service, United States Department of Agriculture, University, MS 38677, USA
[3] Postgraduate Program in Pharmaceutical Sciences, State University of Ponta Grossa, Ponta Grossa, PR 84030-900, Brazil
* Correspondence: dr_aliabbas@hotmail.com or aali@olemiss.edu; Tel.: +1-662-915-3524

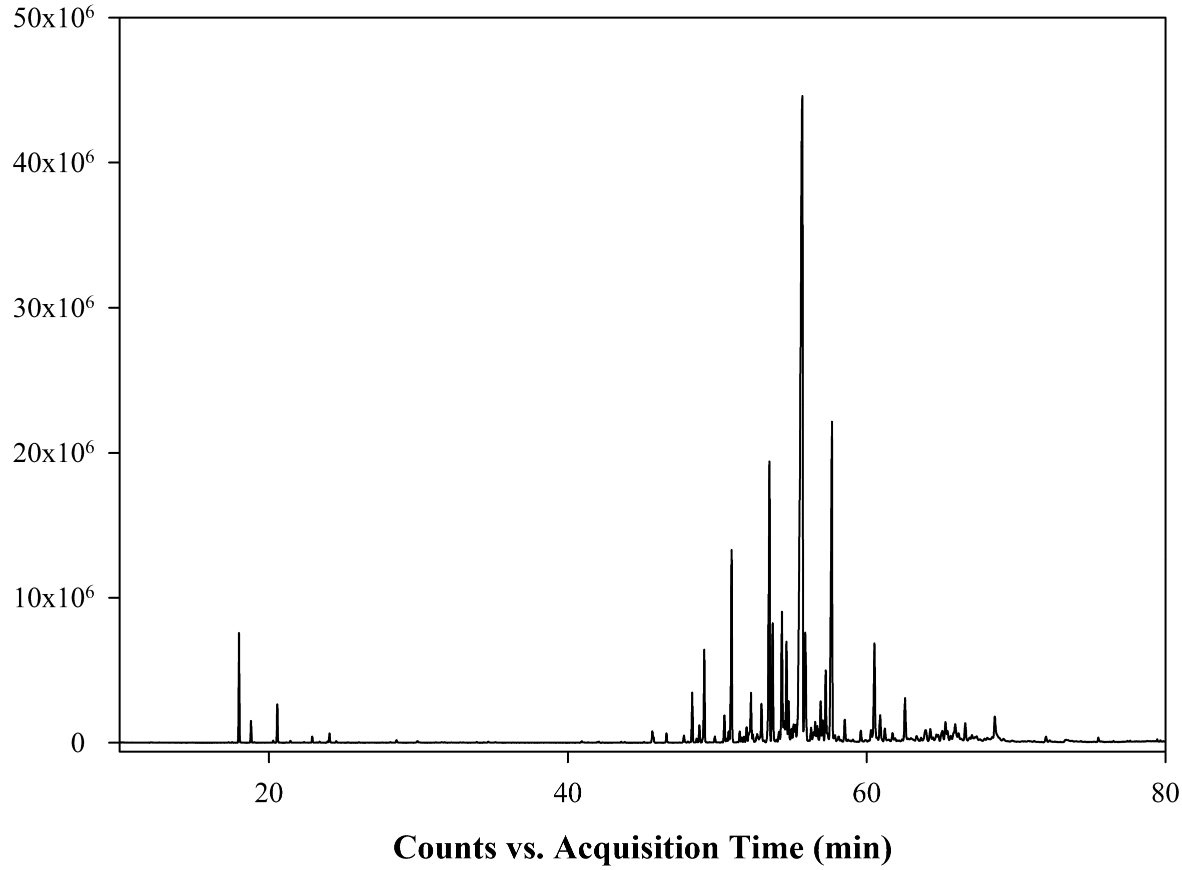

Supplementary Figure S1. GC/MS total ion chromatogram of *P. crassinervium* essential oil.

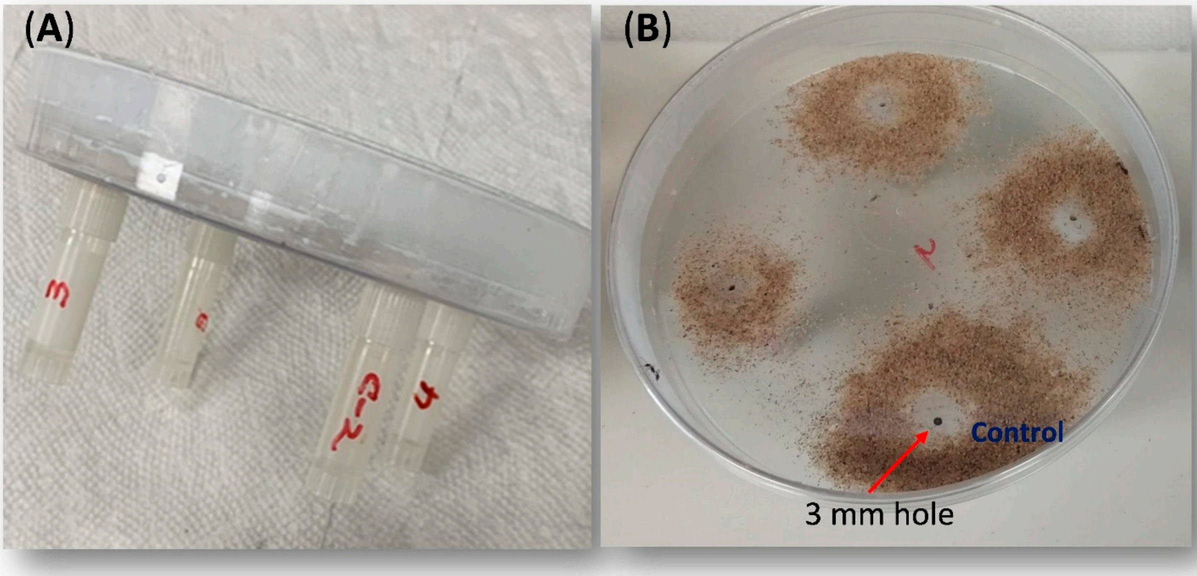

Supplementary Figure S2. Imported fire ants digging bioassay setup **(A)** and digging activity presented as quantity of sand removed as a measure of repellency **(B)**.
